# Impairment of mitochondria dynamics by human A53T α-synuclein and rescue by NAP (davunetide) in a cell model for Parkinson’s disease

**DOI:** 10.1007/s00221-016-4836-9

**Published:** 2016-11-19

**Authors:** T. Q. Melo, K. C. van Zomeren, M. F. R. Ferrari, H. W. G. M. Boddeke, J. C. V. M. Copray

**Affiliations:** 1 Department of Genetics and Evolutionary Biology, Institute for Biosciences, University of Sao Paulo, Sao Paulo, Brazil; 2 Department of Neuroscience, Section Medical Physiology, University Medical Center Groningen, University of Groningen, A. Deusinglaan 1, 9713 AV Groningen, The Netherlands

**Keywords:** Mitochondrial trafficking, α-Synuclein, Mitochondria dysfunction, Neurodegeneration, Parkinson, SHSY5Y, Microtubule, Reactive oxygen species

## Abstract

The formation of oligomers and aggregates of overexpressed or mutant α-synuclein play a role in the degeneration of dopaminergic neurons in Parkinson’s disease by causing dysfunction of mitochondria, reflected in their disturbed mobility and production of ROS. The mode of action and mechanisms underlying this mitochondrial impairment is still unclear. We have induced stable expression of wild-type, A30P or A53T α-synuclein in neuronally differentiated SH-SY5Y neuroblastoma cells and studied anterograde and retrograde mitochondrial trafficking in this cell model for Parkinson’s disease. In contrast to wild-type and A30P, A53T α-synuclein significantly inhibited mitochondrial trafficking, at first retrogradely and in a later stage anterogradely. Accordingly, A53T α-synuclein also caused the highest increase in ROS production in the dysmobilized mitochondria in comparison to wild-type or A30P α-synuclein. Treatment with NAP, the eight amino acid peptide identified as the active component of activity-dependent neuroprotective protein (ADNP), completely annihilated the adverse effects of A53T on mitochondrial dynamics. Our results reveal that A53T α-synuclein (oligomers or aggregates) leads to the inhibition of mitochondrial trafficking, which can be rescued by NAP, suggesting the involvement of microtubule disruption in the pathophysiology of Parkinson’s disease.

**Electronic supplementary material:**

The online version of this article (doi:10.1007/s00221-016-4836-9) contains supplementary material, which is available to authorized users.

## Introduction

Parkinson’s disease (PD) is the most common neurodegenerative movement disorder affecting 1% of people above 65 years of age (Tanner 1992). The pathology of PD is characterized by the degeneration of dopaminergic neurons in the substantia nigra. This neurodegeneration is thought to be associated with the formation of aggregates containing α-synuclein (Siderowf and Stern 2003) and with increased oxidative stress (Qian et al. 2008; Gruden et al. 2011; Simcox et al. 2013). The vast majority of PD patients have the sporadic form of this disease which appears to be age related. A small percentage of PD is familiar and caused by specific mutations. The α-synuclein gene (*SNCA*) was the first gene definitely associated with familiar PD, and there are three known missense point mutations, A53T and A30P and E46 K, besides duplication and triplication of *SNCA*, that all lead to an early disease onset (Polymeropoulos et al. 1997; Kruger et al. 1998; Singleton et al. 2003; Chartier-Harlin et al. 2004). Various genome-wide association (GWAS) studies have shown that SNPs in *SNCA* (and *MAPT*) are also common risk factors for sporadic PD (Edwards et al. 2010; Lill et al. 2012; Nalls et al. 2014).

The molecular mechanisms underlying the neurodegenerative effects of α-synuclein oligomers and aggregates seem to involve most prominently the mitochondria. Although direct damaging effects of α-synuclein oligomers and aggregates on mitochondria have been described, indirect effects on the processes of autophagy and trafficking of mitochondria may be involved as well. Alterations in intracellular degradation pathways, such as macro-autophagy, have been observed in many studies linking protein aggregation mechanisms with neurodegeneration (Victoria and Zurzolo 2015). It has been demonstrated that overexpression of α-synuclein can lead to inhibition of autophagy and concomitant α-synuclein accumulation, whereas the knockdown of α-synuclein resulted in autophagy enhancement, suggesting that α-synuclein may have a regulatory role in autophagy (Winslow et al. 2010) The presence of damaged mitochondria by the direct action of (mutant) α-synuclein oligomers, on the contrary, appears to stimulate excessive mitophagy leading to mitochondrial fragmentation (Wang et al. 2012; Perfeito et al. 2014).

Normal mitochondrial turnover depends on a proper balance between anterograde and retrograde trafficking (Arnold et al. 2011); in PD, this mitochondrial turnover appears to be impaired (Simcox et al. 2013; Hunn et al. 2015). In anterograde trafficking, mitochondria are transported from the soma to the axon up to the synaptic terminals; by retrograde trafficking, the mitochondria return to the cell soma for breakdown and re-entering the biogenesis cycle (Amiri and Hollenbeck 2008). Retardation in anterograde transport can result in an abnormal cellular distribution of mitochondria and a decrease in ATP levels at the synapses (Cai et al. 2005); impairment of retrograde transportation leads to an accumulation of mitochondria in the synaptic terminal interfering with proper synapse formation and function (Van Laar and Berman 2009).

Alpha-synuclein oligomers and aggregates seem to interfere directly with normal mitochondrial turnover (Qian et al. 2008; Celardo et al. 2014). Alterations in axonal transport and in the level of motor proteins have been observed in transgenic *Drosophila* co-expressing tau and α-synuclein, in postmortem brain tissue of sporadic PD patients, in animal and cellular models of sporadic PD and in rats overexpressing α-synuclein (Chu et al. 2012; Chaves et al. 2013; Melo et al. 2013; Roy and Jackson 2014). Studies have shown the interaction between α-synuclein and tau (Credle et al. 2015; Magdalinou et al. 2015). In neurons, tau is essential for stabilizing microtubules and so for enabling proper motor transport (Wade-Martins 2012). In case of overexpression of α-synuclein, tau is phosphorylated, leading to its loss of function and the subsequent impairment of trafficking (Magen et al. 2014; Credle et al. 2015). Esteves et al. (2014) demonstrated that α-synuclein oligomers are able to disrupt microtubules, leading to abnormal axonal trafficking and consequently mitochondrial dysfunction. In particular, the α-synuclein gene mutation A53T is able to form oligomers and aggregates more easily and faster than other types of α-synuclein (Giasson et al. 2002). Accordingly, it was demonstrated that in particular A53T α-synuclein significantly reduced mitochondrial motility in cellular models for PD in which human A53T α-synuclein was expressed, i.e., in mouse hippocampal neurons and SH-SY5Y neuroblastoma cells (Xie and Chung 2012) or in mouse cortical neurons (Li et al. 2013).

Activity-dependent neuroprotective protein (ADNP) is essential for brain formation and provides neuroprotection throughout the entire adult brain; ADNP mRNA and protein expression responds to brain injury and a variety of cytotoxic insults. Structure-activity studies have identified a short eight amino acid peptide in ADNP, NAPVSIPQ (abbreviated to NAP) that appears to be responsible for neuroprotection (Bassan et al. 1999; Zamostiano et al. 2001; Gozes 2007). Treatment with NAP has been shown to restore microtubule integrity and to rescue microtubules-dependent axonal trafficking, and, with that, mitochondrial function (Bassan et al. 1999; Zamostiano et al. 2001; Gozes 2007; Esteves et al. 2014). NAP also contributed to functional recovery in mice overexpressing α-synuclein by reducing hyperphosphorylated tau levels (Magen et al. 2014).

In the present study, we aimed to analyze in more detail the effects of A30P or A53T α-synuclein on anterograde and retrograde mitochondrial trafficking in SH-SY5Y neuroblastoma cells in which we managed to induce a stable expression of wild-type, A30P or A53T α-synuclein. In addition, we have studied the effect of NAP treatment on the mitochondrial mobility and function in these cells.

## Materials and methods

### Cell culture

SH-SY5Y cells (passage 17), obtained from ATCC cell culture, were not used above passage 35 as these cells are reported to lose their neuronal phenotype after repeated passaging. Cells were maintained in DMEM (1×) supplemented with 15% FBS, 1% Pen/Strep, 100 mM Na-pyruvate and 2 mM Glutamax (DFCS) in tissue culture treated dishes or flasks. At 70–80% confluence, cells were passaged using trypsin/EDTA (Lonza) following general cell culture procedures. Split ratios ranging from 1/20 to 1/40 were used to ensure similar densities among transgenic lines. To initiate differentiation into neuron-like cells, SH-SY5Y cells were plated at a density of 2 × 10^4^ cells/cm^2^ in wells pre-treated with poly-d-lysine (PDL). After 1 day, cells were exposed to 10 µM retinoic acid (Sigma) in DMEM (1×) supplemented with 10% FBS for 5 days, after which medium was replaced to DMEM 1× (High Glucose) containing 10 ng/ml BDNF (Peprotech), in order to promote the outgrowth of neural extensions.

### Construction of viral vectors

Viral vectors containing wild-type α-synuclein and mutant α-synuclein A30P or A53T (pENG1-3, a generous gift by Ellen Nollen) were constructed by PCR amplification (Phusion High-Fidelity DNA Polymerase, ThermoScientific) with overhangs for SpeI and NsiI. PCR amplicons and pSin-EF2-Nanog-Pur (Adgene plasmid 16578) were restricted using BcuI (SpeI, Thermo Scientific) and Mph1103I (NsiI, Thermo Scientific), and subsequently ligated using T4 ligase (ThermoScientific) after which the product was transformed in DH5α competent cells. PCR screening was performed to select positive colonies, which were checked by restriction analysis. Correct plasmids were sent for sequencing and transfected in HEK293T to validate expression. Vector constructs are shown in Supplemental Fig. S1.

### SH-SY5Y viral transduction and transfection

Lentiviral particles were generated using a modified protocol based on the protocol of Trono et al. (http://tcf.epfl.ch/page-6764-en.html). Briefly, HEK-293T cells were transfected when cells were 70–80% confluent. A mixture containing 100 µl OPTIMEM (Gibco) 1.4 µg viral vector, 0.4 µg pMD2-VSV-G and 1 µg pCMV-D8.91, was supplemented with 6 µl FUGENE HD (Lonza) and incubated for 15 min at room temperature (RT) to generate transfection complexes. The next day medium was changed with 2 ml OPTIMEM, and viral particles were harvested between 36 and 48 h.

Viral supernatant was collected and sterilized using a 0.45 µm filter (Nalgene), mixed with 10% DFCS in a 1:1 ratio and supplemented with polybrene (8 µg/ml). This mixture was added to a 6-well plate, and 1 × 10^5^ cells were added to be transduced in suspension. The next day media were changed to DFCS 15%, and cells were placed on puromycin (2–4 µg/ml, Sigma) selection three days after transduction. Puromycin selection was continued during cell culture of the lines.

### Characterization of SH-SY5Y by immunofluorescence

Transgenic cell lines cultured on PDL-coated coverslips were fixed in paraformaldehyde 4% for 20 min at RT and rinsed in PBS 3 times. Samples were permeabilized and blocked with PBS containing 0.1% Triton, 1% BSA and 5% normal goat serum for 60 min at RT.

Samples were incubated with rabbit anti MAP2 (1:1000, Abcam), mouse anti α-synuclein (1:500, Invitrogen) or rabbit anti TOM20 (1:1000 Santa Cruz FL-145) antibodies at RT for 1 h followed by incubation of fluorescent secondary antibodies (1:400) and Hoechst for 1 h at RT. Images were acquired using a Leica AF-6000 fluorescent microscope.

### Mitochondria mobility

SH-SY5Y were transfected with pDsRed2-Mito (Clontech Cat. nr: 632421) in OPTIMEM (Gibco) using Lipofectamine 2000 (Invitrogen), following manufacturer’s instructions. After 1 day of transfection, cells were exposed to 200 μg/ml of G418 for 2 weeks in order to select for cell lines containing the plasmid. SH-SY5Y were differentiated as described above, and mitochondrial mobility was measured in live cells after 4, 6 or 8 days of differentiation, using spinning disk confocal microscopy at 63× objective in a climate controlled chamber. The track was calculated by image comparison of the same field every 10 s for 20 min. Single-cell image stacks were analyzed using the ImageJ difference tracker plugin. Kymographs were generated using ImageJ (FIJI) Multi Kymograph plugin. The experiment was repeated three times independently.

### ROS measurements

After 8 days of differentiation, ROS production was measured by incubating cells with CM-H_2_DCFDA probe (Invitrogen) at 10 µM for 1 min. Images were recorded using spinning disk confocal microscopy at a 63× objective, and analyzed using ImageJ. We have used CM-H_2_DCFDA [5-(and-6)-chloromethyl-2′,7′-dichlorodihydrofluorescein diacetate, acetyl ester], an improved, more stable, derivative of DCFDA, as a detector of ROS; this dye is not fluorescent when chemically reduced, but after cellular oxidation and removal of acetate groups by cellular esterases it becomes fluorescent. We measured fluorescence intensity of the transgenic neurons (i.e., expressing A53T, A30P or a surplus of WT α-synuclein) and the control (transfected with an empty vector).

### Mitochondria morphology and connectivity measurement

After 8 days of differentiation, neurons were fixed and stained for mitochondria using TOM20 antibody. Mitochondria morphology and connectivity among fluorescent mitochondria were observed and compared through stained neurons or neurons expressing pDsRed2-Mito. Total amount of neurons containing normal mitochondria morphology was analyzed and quantified.

### Treatment with NAP

NAP (Santa Cruz, sc361778A) was dissolved in ultrapure H_2_O (Sigma Aldrich) at a stock concentration of 5 mM. Cells were treated with 5 nM NAP or vehicle, ultrapure H_2_O, for 24 h to evaluate rescuing of mitochondria trafficking.

### Western blotting

At 8 days of differentiation, protein was extracted using protein lysis buffer. Protein lysates were sonicated and centrifuged at 13,000 rpm for 10 min. The supernatant was fractionated by SDS-PAGE (25 or 50 µg protein/lane) using a 12.5% tris–HCl gel at 100 V for 1.5 h. Proteins were transferred to immobilon membrane (FL-Millipore IPFL 00010) for 1 h at 100 V. Membranes were blocked using PBS with 0.5% Tween 20 and 5% milk for 30 min. The following antibodies were used directed against: β-actin (1:1000, 37 kDa, Abcam, #ab6276) and α-synuclein (1:1000, 14 kDa, Invitrogen, LB509). The blotted protein bands were visualized using Odyssey scanner (Li-Cor Biosciences, Lincoln, NE). Band density was quantified by computer-assisted image analysis software (ImageJ). For reliable quantification of the Western blot data, the defined methodology was followed as described by Taylor et al. (2013).

### Real-time PCR

Total RNA and genomic DNA were collected using an AllPrep DNA/RNA/Protein Mini Kit. CDNA was reverse transcribed from 1 µg of RNA using M-MLV Reverse Transcriptase. GAPDH mRNA and genomic GAPDH were used as reference for normalization. For quantitate real-time PCR, primers were acquired from Biolegio and reactions were run on a Biorad C1000 Touch thermal cycler and analyzed with Biorad CFX manager software.

### Statistical analysis

Mitochondria trafficking, immunofluorescence, Western Blotting and real-time PCR results were analyzed by one-way ANOVA followed by Tukey’s post hoc test. Neurons containing normal mitochondria morphology were analyzed by Student’s *t* test. A *p* value ≤0.05 was considered significantly different, using GraphPad Prism software (GraphPad Software Inc., version 5.00, CA).

## Results

### Characterization of differentiated, α-synuclein transgenic SH-SY5Y cells

**Fig. 1:**
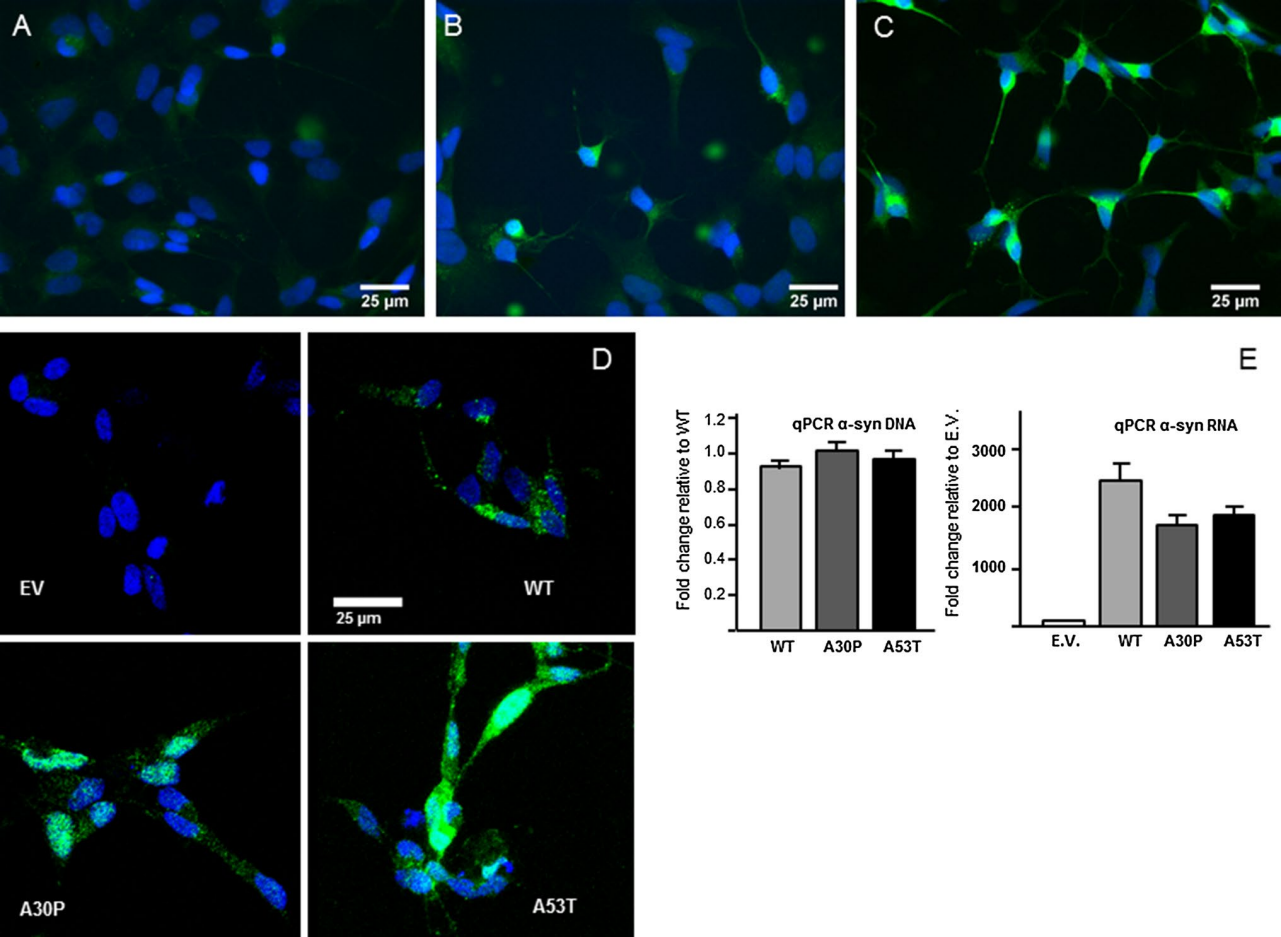
Characterization of differentiated, α-synuclein transgenic SH-SY5Y cells. Immunofluorescence shows a positive stain for microtubule associated protein (MAP2) (*green*) at 4 days of differentiation (**a**), at 6 days of differentiation (**b**) and at 8 days of differentiation (**c**). Immunostaining for α-synuclein shows its expression in the α-synuclein transgenic SH-SY5Y cells (WT, A30P and A53T) but not in the control cells (transfected with an empty vector, EV) (**d**). *Blue* staining in **a**–**d** is Hoechst nuclear staining. Quantification of α-synuclein with q-PCR after 8 days of differentiation confirms the increased expression of α-synuclein (**e**)

After 4 days in vitro (DIV) in the presence of retinoic acid and BDNF, the transgenic SH-SY5Y cells (i.e., expressing A53T, A30P or a surplus of WT α-synuclein) and the control SH-SY5Y cells (transfected with an empty vector) differentiated into neuron-like cells and formed small neurite-like extensions; at 6 DIV, these cells started to express the neuronal marker MAP2 and continued to grow their neurite-like extensions reaching stable maturity at 8 DIV (Fig. 1a–c). No differences in the neuronal differentiation pattern were observed between the control cells and the ones expressing the α-synuclein variants (supplemental Fig. 2). Selective survival based on puromycin resistance resulted in a neuronally differentiated SH-SY5Y cell culture of which all cells expressed a high level either of A53T, A30P or WT α-synuclein; in the control SH-SY5Y cell cultures, expression of α-synuclein could not be detected through immunofluorescence (Fig. 1d). The expression of the α-synuclein variants in the differentiated transgenic and control SH-SY5Y cells was quantified at 8 DIV using qPCR (Fig. 1e). Results of qPCR showed that, although each of the transgenic cell lines contained a similar number of α-synuclein gene copies, transcriptional activity was lower in the cell lines containing mutant α-synuclein in comparison to those expressing wild-type α-synuclein, suggesting interference of these α-synuclein mutants with transcription, nuclear shuttling, or cytoplasmic mRNA processing.

**Fig. 2:**
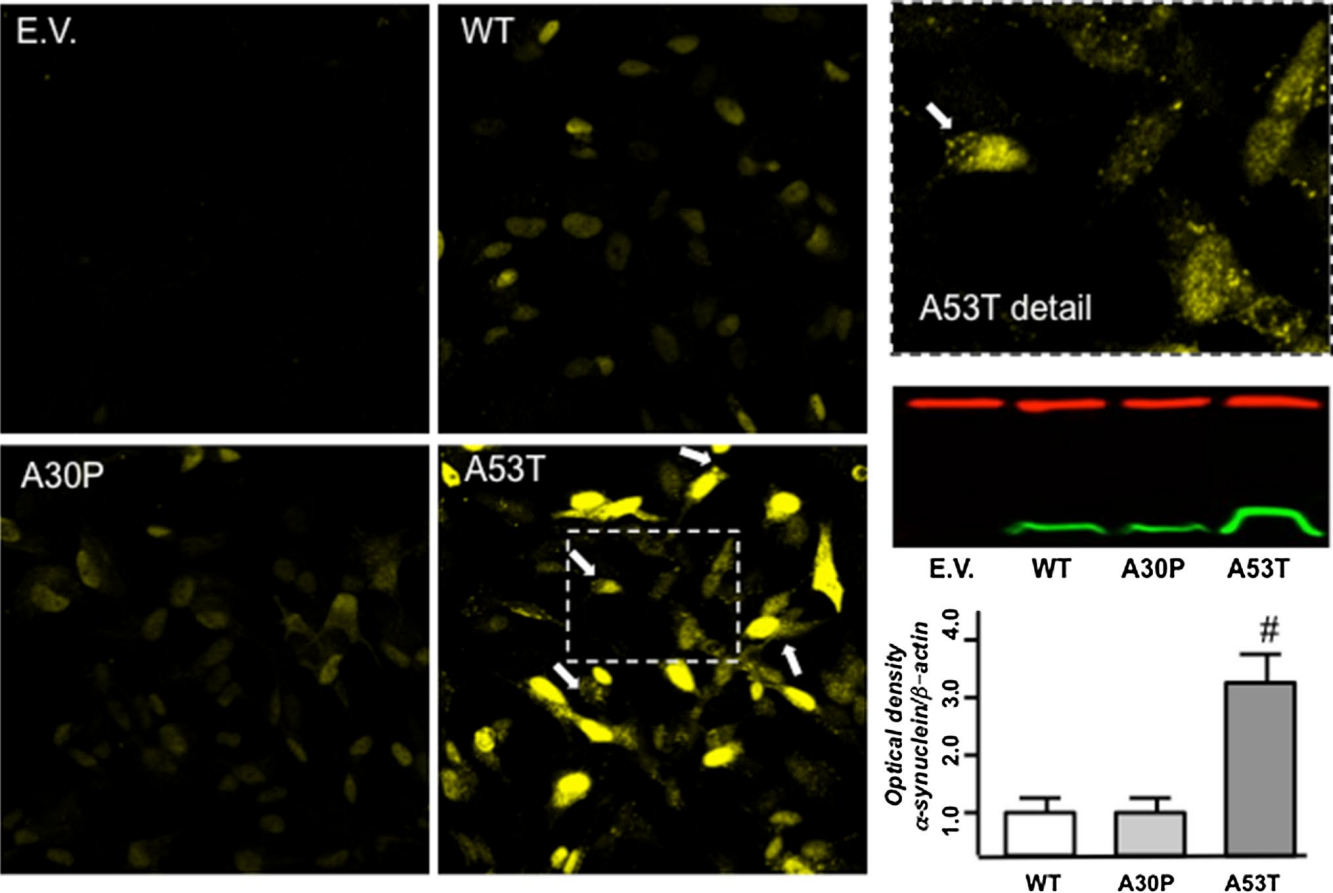
α-Synuclein expression. Photomicrographs show homogeneous α-synuclein staining in neurons expressing WT or A30P YFP-α-synuclein. Neurons expressing A53T YFP-α-synuclein showed puncta accumulation of the protein at soma and neurites (*arrows* in magnification of *boxed area*). Blottings of insoluble protein fraction (*red bands* = β-actin; *green bands* = α-synuclein) reveal significantly higher levels of A53T α-synuclein compared to WT and A30P α-synuclein. Experiments were repeated 3 times. ^#^ *p* < 0.001 as compared with WT according to one-way analysis of variance (ANOVA) followed by Tukey posttest

Analysis of α-synuclein linked to YFP corroborated the results of immunofluorescence, suggesting that A53T is more prone to aggregation since its pattern of labeling resembles that of small aggregates (Fig. 2). The expression of α-synuclein was quantified in Western blots demonstrating increased expression of A53T isoforms (Fig. 2). Our findings suggest a reduction in degradation, possibly due to the aggregated state of A53T α-synuclein.

### Mitochondrial trafficking in differentiated, α-synuclein transgenic SH-SY5Y cells

**Fig. 3:**
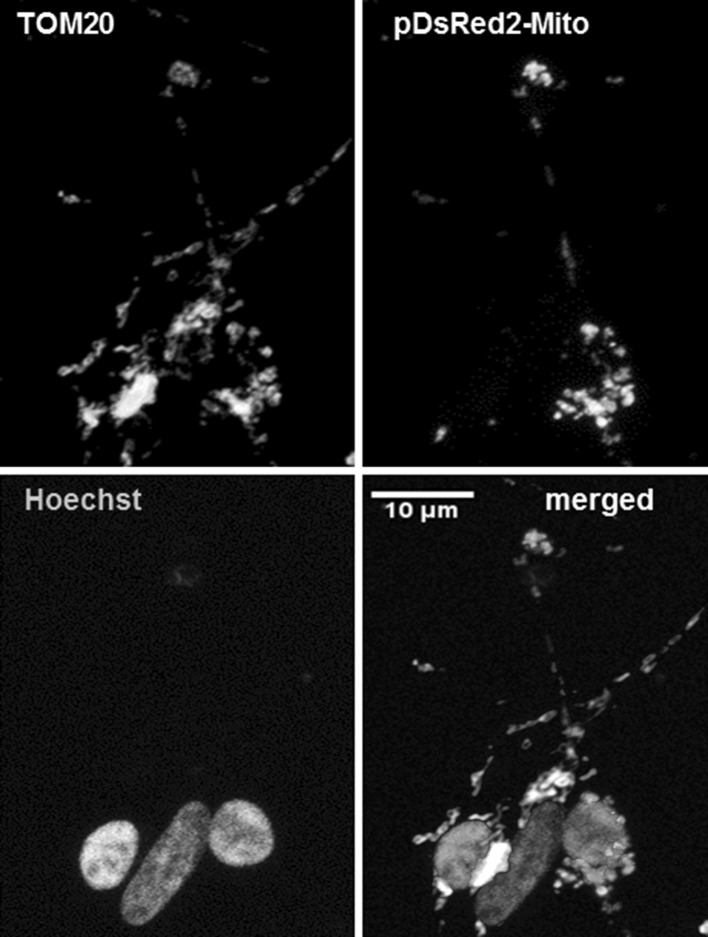
Mitochondrial labeling. Immunofluorescence imaging shows the co-localization of fluorescent staining for the mitochondria-specific TOM20 (*green*) and pDsRed2-Mito (*red*). In the merged picture, the nuclei are *blue* due to the Hoechst nuclear staining

We transfected the various neuronally differentiated SH-SY5Y cell cultures with the pDsRed2-Mito plasmid: approximately 50% of the cells showed red staining in the cytosol that appeared to be confined to mitochondria and completely co-localized with the green immunostaining by the mitochondria-specific TOM20 antibody (Fig. 3). The strong specific staining by pDsRed2-Mito allowed us to investigate mitochondrial mobility in the neurite-like extensions in the neuronally differentiated SH-SY5Y cells and the consequences of aberrant α-synuclein expression for mitochondrial trafficking.

**Fig. 4:**
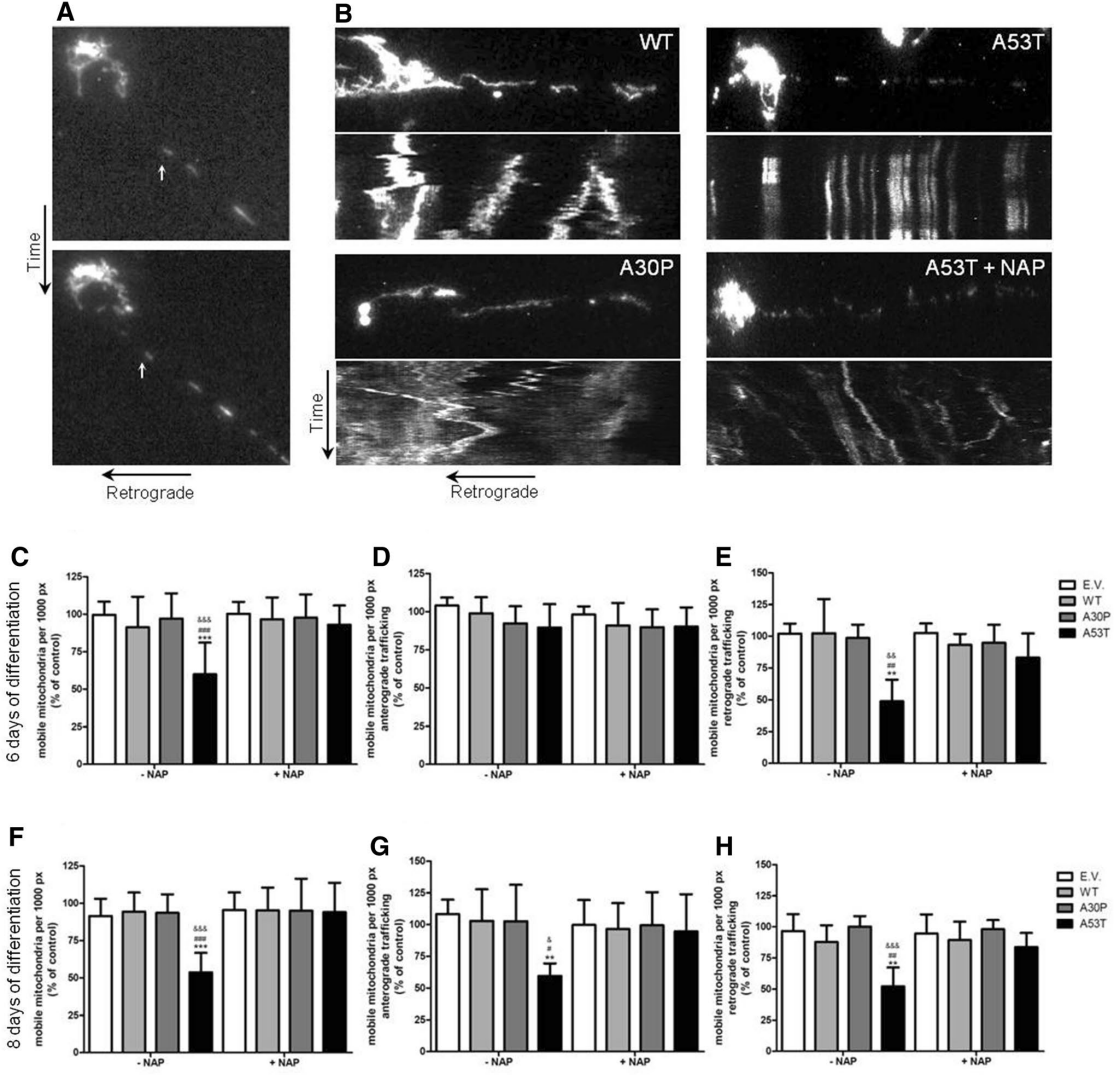
Mitochondria mobility analysis in SH-SY5Y. **a** Time-lapse recordings show the movements of mitochondria retrogradely in the neurite-like extension of a SH-SY5Y cell after 8 days of differentiation. **b** Kymographs represent mitochondria movement in neurons derived from SH-SY5Y cells after 6 days of differentiation expressing either wild-type, A30P or A53T α-synuclein (in the absence and presence of NAP). Analysis of movement reveals that trafficking is decreased at 6 and 8 days of differentiation (**c**, **f**, respectively). Separate analysis of retrograde and anterograde trafficking shows unaltered anterograde trafficking but decreased retrograde trafficking at 6 days of differentiation (**d**, **e**); at 8 days of differentiation both anterograde and retrograde trafficking appears to be reduced (**g** and **h**, respectively). Neurons treated with NAP at 5 nM for 48 h show a rescued mitochondria trafficking at 6 and 8 days of differentiation (**c**, **e**–**h**). Data of moving mitochondria per 1000 pixels are expressed as percent relative to control (E.V.) ± SD. Two-way ANOVA following Bonferroni posttest were the statistical tests employed *p ≤ 0.05 compared to control. ^#^ *p* ≤ 0.05 compared to cells expressing WT. ^&^ *p* ≤ 0.05, compared to cells expressing A30P α-synuclein. Data are expressed as mean of three independent experiments

In order to examine if and when mitochondria traffic was impaired by α-synuclein in SH-SY5Y cell cultures, we analyzed mitochondria mobility in the different transgenic and control SH-SY5Y cells at 4, 6 and 8 DIV in the presence of retinoic acid and BDNF representing different stages in differentiation. At 4 DIV, when the neuron-like cells were still immature and proper mitochondria trafficking was crucial for the outgrowth of the young “neurites,” a similar pattern of mitochondrial trafficking activity was observed in all the groups of SH-SY5Y-derived neuron-like cells. The first sign of disturbed mitochondrial mobility was observed at 6 DIV only in the A53T α-synuclein expressing SH-SY5Y cells where retrograde trafficking was significantly decreased in comparison to the control. Only later, at 8 DIV, also the anterograde trafficking of mitochondria was significantly decreased to almost 30%, again only, in the A53T α-synuclein expressing SH-SY5Y derived cells. No changes were observed in the other transgenic SH-SY5Y cell lines or in the control cells (Fig. 4). So, Fig. 4 presents evidence for the presence of mitochondria trafficking during differentiation and shows that the disturbance in mitochondria trafficking is specific for neuronal cells, since it does not occur in undifferentiated SH-SY5Y cells.

### Effect of NAP on mitochondrial trafficking

To examine the mechanisms by which the A53T α-synuclein-induced impairment of mitochondrial trafficking in the neuronally differentiated SH-SY5Y cells, we treated the SH-SY5Y cell cultures with NAP since it has been shown to restore microtubule integrity (Gozes 2007; Esteves et al. 2014). Adding NAP, indeed, appeared to completely restore retrograde as well as anterograde mitochondria trafficking in the A53T α-synuclein expressing SH-SY5Y derived cells (Fig. 4). Although both motor proteins, dynein and kinesin, have clear structural distinctions and wander across the microtubule surface with different speed, step sizes and in opposite directions, apparently the A53T α-synuclein-induced microtubules destruction, restorable by NAP, affects the interaction between the motor proteins and the microtubules in a similar mode.

### Effect of NAP on mitochondrial membrane potential and morphology

Impairment of mitochondrial dynamics due to the overexpression of α-synuclein or the expression of its mutants A30P and A53T may involve an increase in the production ROS and a disruption of the mitochondrial membrane potential.

**Fig. 5:**
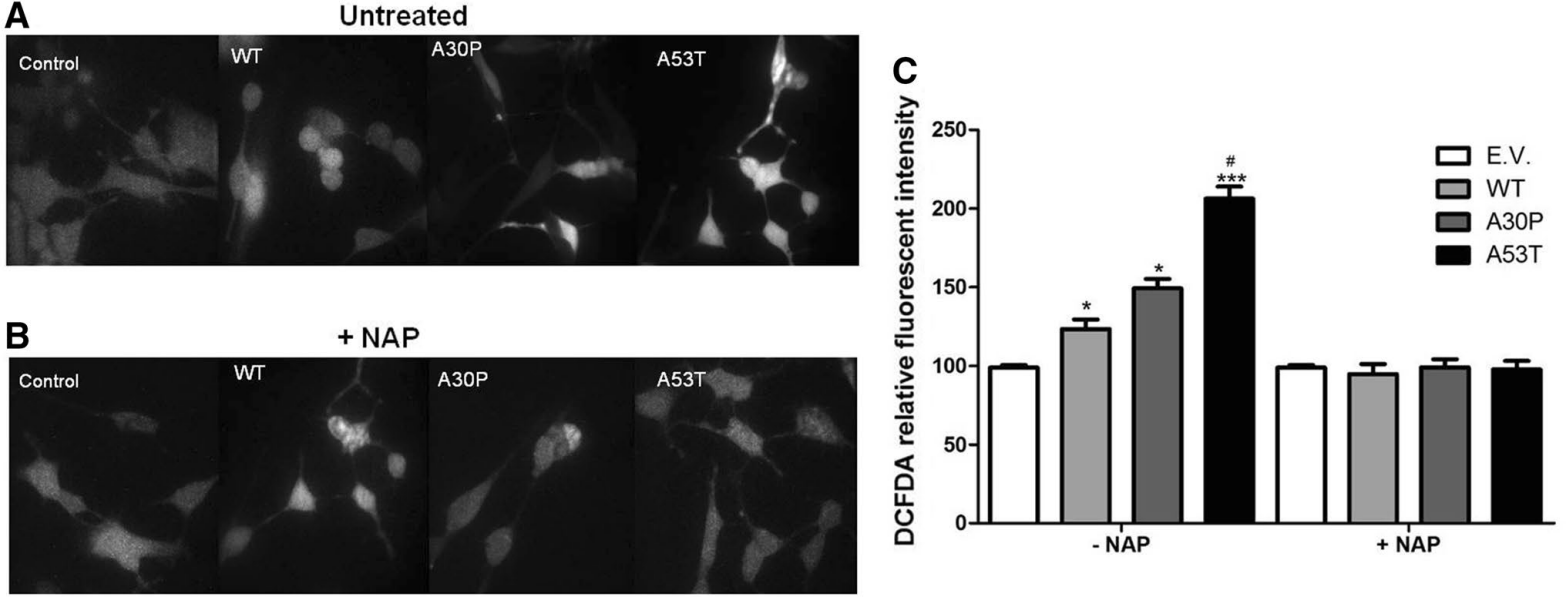
ROS production. Photomicrographs show neurons incubated with green fluorescent probe DCFDA untreated (**a**) or treated with NAP (**b**). Differentiated SH-SY5Y cells at 8 DIV expressing WT, A30P or A53T α-synuclein or controls (E.V. = empty vector) were incubated with CM-H_2_DCFDA, a fluorescent ROS detector, untreated (**a**) or treated with NAP (**b**). Fluorescence intensity measurements (**c**) show significantly higher levels of ROS compared to control (E.V.); treatment with NAP restored levels of ROS to basal levels of control cells. Data are expressed as percentage of control (E.V.) ± SD. Two-way ANOVA following Bonferroni posttest was the statistical test employed *p ≤ 0.05; **p ≤ 0.01; ***p ≤ 0.001 compared to control. ^#^ *p* ≤ 0.05 compared to cells expressing WT. Data are expressed as mean of three independent experiments

We found a significant increase in ROS levels in the α-synuclein expressing cells in comparison to the controls, with the highest level in the A53T mutant (Fig. 5a, c). Adding NAP to the culture, completely annihilated the ROS increase observed in all the α-synuclein expressing cell lines (Fig. 5b, c).

**Fig. 6:**
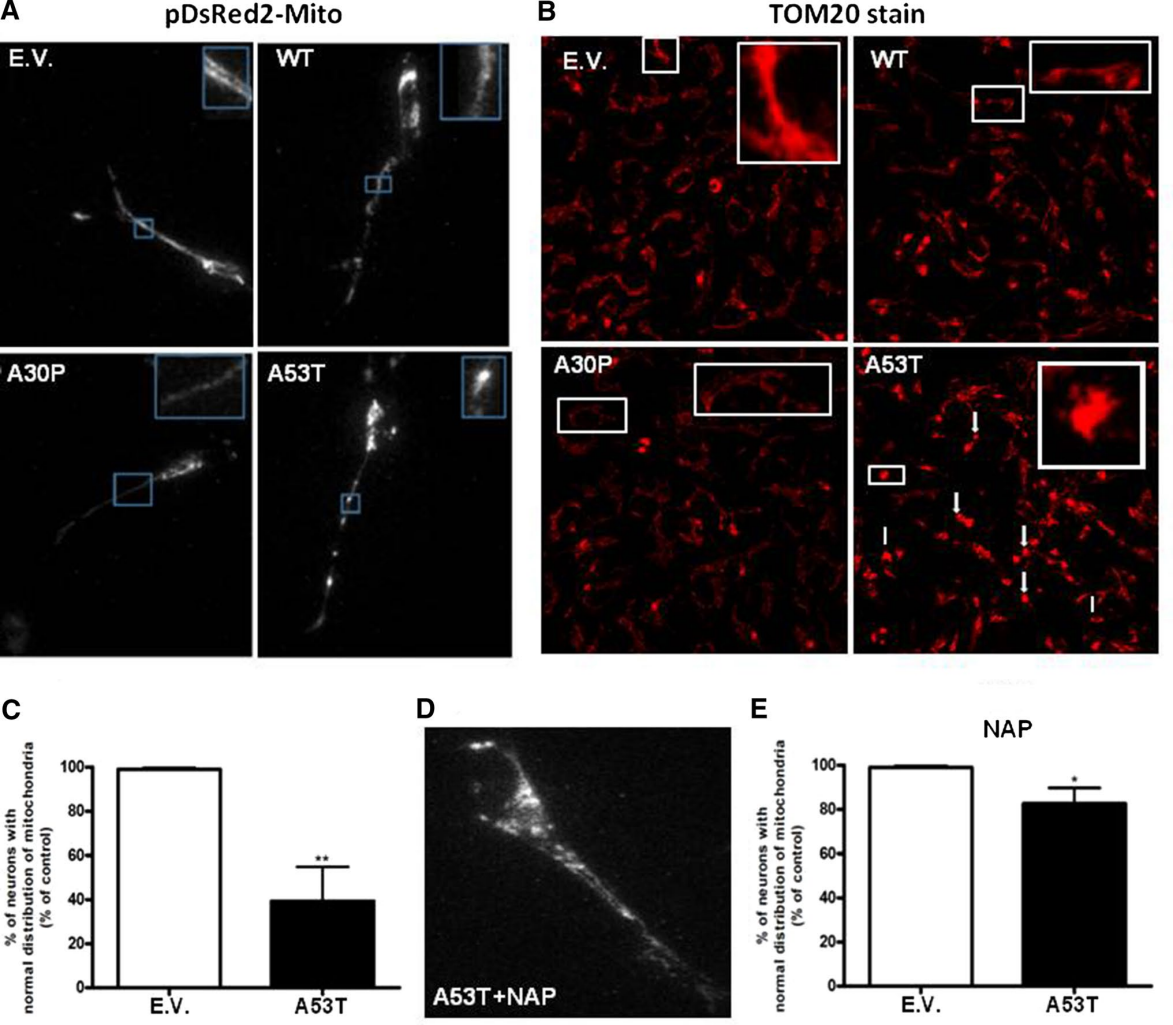
Mitochondria distribution and connectivity. Photomicrographs illustrate mitochondria distribution and connectivity in neurons transfected with pDsRed2-Mito (63× objective) (**a**) or in neurons stained for mitochondria using TOM20 antibody (20× objective) (**b**). Quantification of neurons with normal mitochondria distribution reveals a significant decrease after expression of A53T α-synuclein (**c**). Treatment with NAP increases the amount of neurons with normal mitochondria distribution and connectivity to 70% (**d** and **e**). Data are expressed as percent relative to control (EV). *p < 0.05 and **p < 0.01 compared with EV according to Student’s *t* test. *n* = 5. Experiments were repeated three times

Living neuronal cells transfected with pDsRed2-Mito or those fixed and stained for TOM20 showed the same pattern of mitochondria morphology when expressing WT or A30P alpha-synuclein compared to control (transfected with empty vector). About 65% of neurons expressing A53T alpha-synuclein showed small spherical mitochondria clusters. After NAP treatment, only 20% of DA neurons expressing A53T alpha-synuclein showed fragmented mitochondria (Fig. 6).

## Discussion

Our studies show that, in contrast to wild-type and A30P α-synuclein, A53T α-synuclein significantly inhibited mitochondrial trafficking in the SH-SY5Y cell model for Parkinson’s disease. Retrograde trafficking appears to be the first to be disturbed, followed in a later stage by anterograde trafficking. Accordingly, A53T α-synuclein also caused the highest increase in ROS production in these apparently dysmobilized and fragmented mitochondria in comparison to wild-type or A30P α-synuclein. Treatment with the active peptide (NAP) of activity-dependent neuroprotective protein (ADNP) completely annihilated the adverse effects of A53T on mitochondrial dynamics.

Our findings regarding the effect of A53T α-synuclein on mitochondrial trafficking not only confirm but also extend previous data (Xie and Chung 2012; Li et al. 2013) since we also studied the time frame of mitochondria trafficking disturbance: we wanted to determine which type of trafficking was disturbed first, anterograde or retrograde. Impairment of anterograde or retrograde trafficking can lead to different injuries in organelles in neurons. In our previous studies (Chaves et al. 2013; Melo et al. 2013), we revealed that the expression of proteins involved in anterograde or retrograde trafficking was differentially affected. It has been reported that disrupted anterograde mitochondria trafficking leads to fragmentation of mitochondria, abnormal mitochondria distribution and biogenesis, depletion of ATP and high ROS levels, resulting in cell death (Matenia et al. 2012), while disrupted retrograde trafficking leads to aging and swollen mitochondria, depletion of ATP, higher ROS levels and cell death (Morris and Hollenbeck 1993). Although the outcome (cell death) is the same, determining the direction of trafficking that is impaired first enables elucidation of the actual mechanisms responsible for neuronal death; it may lead to a more specific therapeutical approach to prevent the phenotype of the disease. In the present study, we showed that A53T first impairs retrograde trafficking in neurons derived from the SHSY-5Y cells. In addition, we showed that NAP is able to recover mitochondria trafficking.

The prominent effect on mitochondrial trafficking of A53T α-synuclein in comparison to A30P and wild-type α-synuclein most likely reflects the fact that A53T α-synuclein is more prone for oligomer or aggregate formation. It has been shown that the interaction of wild-type α-synuclein and α-synuclein variants with molecular motors, tubulin, and the microtubules-associated proteins, MAP2 and Tau, is stronger for oligomers than for monomers (Prots et al. 2013). There appears to be differential effects between seeds and oligomers on (Tau-promoted) microtubules assembly and on the microtubules gliding velocity across kinesin-coated surfaces (Prots et al. 2013). Due to the fact that NAP, a protein known to repair microtubules integrity, restores the A53T α-synuclein-induced impairment of mitochondrial trafficking, may point to a disruption of microtubules assembly by A53T α-synuclein disturbing the interplay between microtubules and kinesin (Prots et al. 2013). The earliest effects of A53T α-synuclein was observed in the retrograde dynein-driven trafficking of mitochondria, indicating that also the dynein-microtubules interplay may be disturbed. The reason that retrograde mitochondrial trafficking is the first to be affected may be related to the lower velocity of retrograde transport in which even small disturbances leads to an actual stop in trafficking. Obviously, the machinery underlying mitochondrial trafficking along the microtubules in axons concerns a complex structure consisting, besides of kinesin and dynein, of Miro (also known as RhoT1/2) and Milton (for a review see Schwarz, 2013). Interactions between oligomers/aggregates of α-synuclein and Miro and Milton are as yet unknown.

Although the best known action of NAP is upon microtubule stabilization, this peptide has also been reported to affect autophagy (Gozes 2016) and oxidative stress (Greggio et al. 2011; Sharma et al. 2011). In fact, we observed a decrease in ROS content after NAP treatment, which may be either the cause or the consequence of altered mitochondria trafficking.

A wide variety of studies have shown that changes in mitochondrial trafficking are the earliest events to occur in cell models for Parkinson’s disease with abnormal α-synuclein expression (Coskun et al. 2012; Prots et al. 2013; Arduino et al. 2015; Keogh and Chinnery 2015; Franco-Iborra et al. 2016). The stressed, dysmobilized, mitochondria start to produce high levels of ROS, subsequently leading to other cytopathological processes. The annihilating effect of NAP on the ROS levels in our cell cultures expressing α-synuclein variants must be ascribed to the recovery of the mitochondrial motility by NAP (Esteves et al. 2014).

Summarizing, we propose that A53T α-synuclein leads to impairment of trafficking in SH-SY5Y cells, most likely by a disturbance of microtubule integrity. This in turn leads to impairment of mitochondrial turnover, particularly in distal regions of the cell. NAP treatment rescues mitochondria trafficking and so proper mitochondrial function and turnover. After 8 days of differentiation of the SH-SY5Y cells, we observed aggregates only in neurons expressing A53T alpha-synuclein. So, likely we have oligomers of WT, A30P and A53T alpha-synuclein after 6 days of differentiation and of WT and A30P after 8 days of differentiation. As cited above, time to form oligomers of alpha-synuclein is dependent of its type, where A53T alpha-synuclein oligomerizes easier and faster than A30P alpha-synuclein, which in turn oligomerizes easier and faster than WT alpha-synuclein. Moreover, not only the levels of oligomers influence cellular damage, but also the type of oligomer is important for the type and amount of damage in neurons (Stefanovic et al. 2015). In view of this, our findings suggest that we have more oligomers of A53T alpha-synuclein and that they are more toxic than oligomers of A30P or WT alpha-synuclein. The increase in ROS production we observed may be related with lowered mitochondrial integrity at distal sites. We have shown that these effects can be rescued by using NAP, negating the effect of α-synuclein expression and leading to increased mitochondrial turnover.

Our data implies mitochondrial trafficking as an important mechanism relating mitochondrial damage to protein occlusions observed in Parkinson’s disease. Early impairment of retrograde transport would lead to accumulation of damaged mitochondria near the axon terminal, which has been proposed as an early event in clinical cases of Parkinson’s disease (Cheng et al. 2010).

## Electronic supplementary material

Below is the link to the electronic supplementary material.

**Supplemental Fig.** **1**. Example of the composition of the lentiviral vector used for the transfection of the α-synuclein (trans) genes (TIFF 37 kb)

**Supplemental Fig.** **2**. Neuronal-like differentiation of the various SH-SY5Y cell lines at 4, 6 and 8 days in vitro (DIV). No apparent differences in differentiation pattern were observed between the different cell lines. Phase-contrast microscopy, *bar* 50 µm (TIFF 336 kb)

Supplementary material 3 (DOCX 13 kb)
