# Supplementary figures and images for: Impairment of mitochondria dynamics by human A53T α-synuclein and rescue by NAP (davunetide) in a cell model for Parkinson’s disease
Source: Exp Brain Res. 2016 Nov 19;235(3):731–42. doi: 10.1007/s00221-016-4836-9 (PMC5315729; doi:10.1007/s00221-016-4836-9)

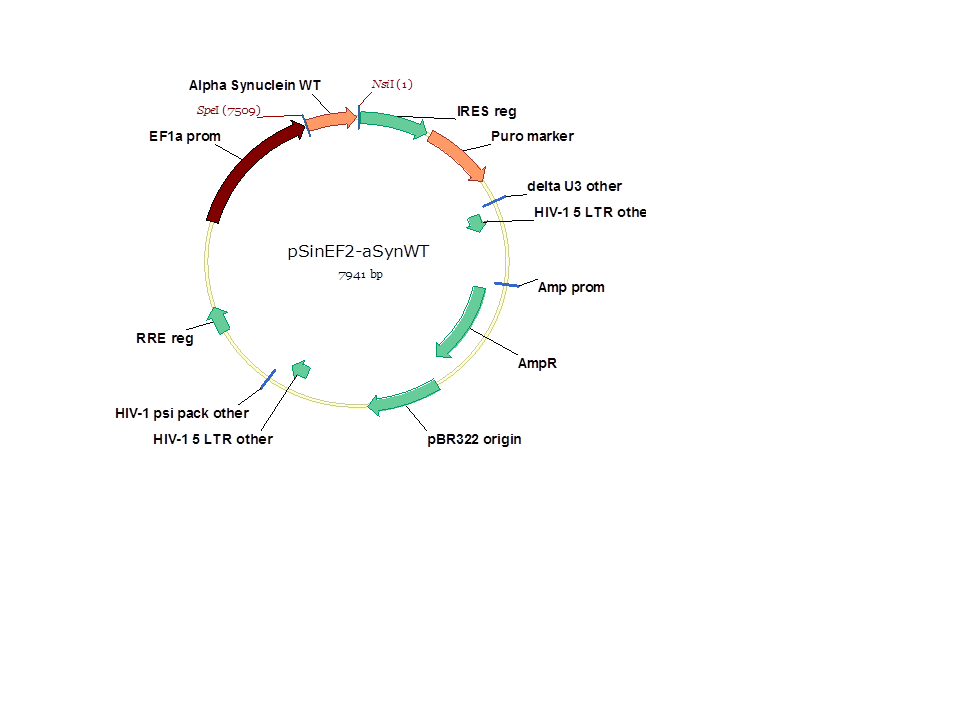

Supplement: Supplementary file 1 — Supplemental Fig. 1. Example of the composition of the lentiviral vector used for the transfection of the α-synuclein (trans) genes (TIFF 37 kb) [file 221_2016_4836_MOESM1_ESM.tif]

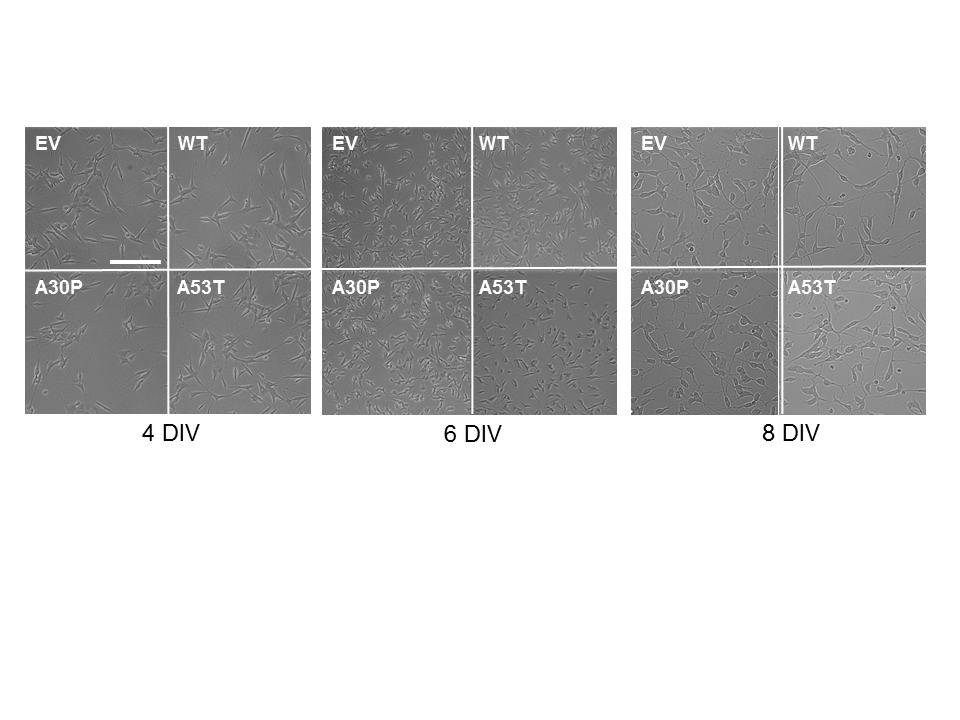

Supplement: Supplementary file 2 — Supplemental Fig. 2. Neuronal-like differentiation of the various SH-SY5Y cell lines at 4, 6 and 8 days in vitro (DIV). No apparent differences in differentiation pattern were observed between the different cell lines. Phase-contrast microscopy, bar 50 µm (TIFF 336 kb) [file 221_2016_4836_MOESM2_ESM.tif]
