# Supplementary material for: Impairment of mitochondria dynamics by human A53T α-synuclein and rescue by NAP (davunetide) in a cell model for Parkinson’s disease
Source: Exp Brain Res. 2016 Nov 19;235(3):731–42. doi: 10.1007/s00221-016-4836-9 (PMC5315729; doi:10.1007/s00221-016-4836-9)
Supplement: Supplementary file 3 — Supplementary material 3 (DOCX 13 kb) [file 221_2016_4836_MOESM3_ESM.docx]

Supplemental Table 1. List of primers used.

| Target | Fw. Primer | Rev. Primer |
| --- | --- | --- |
| GAPDH | TTTCTATAAATTGAGCCCGCAGC | TACGACCAAATCCGTTGACTCC |
| GAPDH DNA | AACCTGCCAAATATGATGACATCA | AGCCCAGGATGCCTTTGAG |
| α-synuclein | AAGAGGGTGTTCTCTATGTAGGC | GCTCCTCCAACATTTGTCACTT |
| Plasmid α-synuclein | AACTAGTATGGATGTATTCATGAAAGGAC | AATGCATTTAGGCTTCAGGTTCGTAG |
| Seq. Primer EF1a | TCAAGCCTCAGACAGTGGTTC |  |
